# Meningitis

**Published:** 1891-04

**Authors:** J. C. Michener


THE JOURNAL

OF

COMPARATIVE MEDICINE AND

VETERINARY ARCHIVES.

VOL. XII. APRIL, 1891. NO. 4.

### MENINGITIS.*

BY J. C. MICHENER, M. D., V. S.

Since our last meeting, I received notice to prepare a paper upon Meningitis. According to Prof. Williams, this is a disease of rare occurence, in veterinary practice; which coincides with my experience. But I took it for granted, that it was intended that I should take up the more fruitful theme known to our profession as Cerebro Spinal Meningitis, and to the non-professional, by such local names as Choking Distemper, Putrid Fever, Putrid Sore Throat, Paralysis, Falling Sickness, Corn stalk disease, and the Jersey disease," all of which nomenclature is defective inasmuch as it fails to denote either the pathology, or cause of the malady; the professional name being the worst of the whole lot. Even to-day I shall not venture a name for fear time will show it to need remodeling like the rest. Although we have very little literature upon the subject, and it is generally regarded as a new disease, it has been known, and dreaded, in our district by the oldest men and their predecessors. My father has been handling the disease the past fifty years and his preceptor had experience with it for a number of years before that.

It has such an extended range of symptoms, owing to the many different parts of the nervous system upon which it may concentrate its force, and the extreme variability in the severity

*Read before the Pennsylvania State Veterinary Medical Association March 3, 1891.

of the attack, that the practitioner must be well-versed in the whole line of symptoms and in differential diagnosis, or many cases will go unrecognized, and others will be allowed to advance to the last fatal stages before the gravity of the case is realized. Therefore, I trust, you will bear with me while giving the symptoms minutely, and we will then better understand each other in the discussion of the subject.

The general and first symptoms noticed are those of muscular paralysis, more or less complete and more or less slowly induced, according to the severity of the attack and varying considerably in the parts principally affected. This nervous depression seems to be a surrender of the vital principle, and the chemical changes of decomposition of death commence, and in protracted cases progress to the point of putrefaction while the animal is yet alive. We will first consider the worst type of the disease, then milder attacks, on down the grade, until it takes the keen practical observer to detect its existence.

I will narrate three cases as they occurred representing the disease in its greatest severity. Mr. Samuel Aaron, of Hillton, worked his three horses one day without noticing anything amiss, they ate as well and appeared as strong and active as usual. At four o'clock the next morning he was aroused by a thumping noise at the barn, and on going to the stable found a horse down flat on his side, and every few minutes striking violently with all four feet at once, making the splinters fly out of a strong partition. Procuring help the horse was dragged out of the stall, turned upon his breast, and strong efforts were made to get him upon his feet but he could not even hold his head up, and fell flat on his side the instant the helpers let loose their hold. Gearing one of his other horses he drove for me, the animal performing quite well. He turned back and I followed in a few minutes; after going three miles, I overtook my customer walking and leading his horse which was scarcely able to drag himself along, sweating and panting. We changed teams. I took the sick horse from the buggy and taking a short hold by the head, led him very carefully, bracing him at the shoulder, and urging him up whenever I felt his knees giving way. In about half an hour we accomplished the remaining mile and placed him at once in slings, as he was sweating profusely and trembling (Substultus Tendinum). The pulse was peculiar and characteristic, being rapid and intermitting, stopping entirely for an instant from time to time. After being rubbed and rested a few minutes the pulse dropped to thirty-two missing about two beats after making ten or twelve, respiration natural. I had a bucket of water brought, the poor fellow was very anxious to drink, thrusting the nose deeply in and contracting the facial muscles and sterno maxillaris in a peculiar manner, as if determined to force some down, making a characteristic gulping sound, that would lead an inexperienced person to suppose he was drinking very fast, when in reality he was not getting a drop, but willing to persist in trying. The water being taken away and feed brought, he took some with the lips and vainly tried to work it up into the mouth, the saliva coming quite freely. The horse found down at four o'clock had by this time, seven o'clock, died.

We now directed our attention to the third horse, he had eaten his breakfast up clean, and the spectators pronounced him all right. I directed him brought out of the stable, he blundered over the door sill and fell down, unable to rise of himself but was gotten upon his feet by hand-help; a bucket of water was brought which he drank, taking him fully twice as long as it should, the water coming down the nostrils in streams the thickness of rye straws. The pulse, respiration, and temperature normal, eyes bright, and countenance cheerful. I tied his head up short and gave him hay which he chewed and swallowed slowly. Now we turned our attention to horse No. 2, he was hanging in the slings like a wet dish-cloth; let him down and he was dead by nine o'clock. The first sickened about six o'clock, the third and last horse fell and broke his halter at half-past nine and was dead by ten o'clock. Each horse died in three hours after being found sick. I have known a few others to die in from six to twelve hours and a great many to die in from twenty-four to thirty-six hours. When dying thus early they seem to do so quite easily, and unexpectedly to the attendants, but those cases that linger from four to five days on to two or three weeks, are apt to live many hours after they are expected to die. In many of the first named cases they die of nervous shock without leaving a single pathological change visible to the naked eye.

I will picture to your minds a stable of six horses having as many forms of the disease in question.

First, the typical case. He is noticed to be unable to work as usual, walks slower, sweats early and stubs his toes often. His gait is peculiar and diagnostic. The muscles have lost their accustomed elasticity, he does not pick his feet up as high as usual and gives them a rotary motion, planting them more beneath the body, having more motion in the shoulders than natural. If it be fly time, he is greatly annoyed, he has lost the use of the *panniculus carnosus* and cannot shake the skin, he has not the full use of the tail and can only bring it partly around in a feeble way, wants to stop often and throw the head around at the flies, striking the body with the mouth, and leaving a wet spot on the hair from the saliva that is constantly dribling from the mouth. This is very noticable when feeding upon grass, or a stickey mash, his sides being covered with green or mealy spots. His voice is very feeble, if it be a mare suckling a foal, it is painful to hear her attempting to call the colt unable to raise her voice above a faint call. In the stable the subject soon has the manger saturated with a thick ropy saliva, and pellets of hay that he has dropped, unable to swallow them. The manner of drinking or attempting to drink is diagnostic. The horse will persist in trying for a long while, keeping steadly at it, but getting his water very slowly, most of it returning back by the nose, never wincing, except occasionally he may stop and give a spasmodic cough as if choked, which is really the case; hence the name Choking Distemper. Usually, in from three to five days he goes down, flat on the side, unable to turn upon his breast without assistance, he soon becomes restless and attempts to get up but his head drops again with a heavy thud. Soon he has a great hole cut in the ground with his hoofs, striking violently with all four at once and always hitting the same spot, unable to get away from where he first spread himself out. Soon he has himself worn sore in many places. Run the hand in on top of the tongue, and when removed, it will have a sickening stench, hence the name putrid fever; coupled with the inability to swallow, it is sometimes called putrid sore throat; but there is never a trace of soreness or inflammation. The urine trickles from the bladder, hence the mare shows signs of oestrum from irritation of the clitoris. The fæces are voided in large masses with much effort, showing a weakening relaxed condition of the muscular walls of the intestines. Before the horse has gone down there will be noticed a rapid wasting of flesh, the abdomen is tucked up and the tendons standing out prominently. In the early stages of the disease if the pulse be taken, when at rest, it will be found normal or a little slower; but move or excite the patient and it becomes rapid and intermitting. There is great weakness. Temperature normal or low. In rare instances there is elevation of temperature in the last stages. After the patient has gone down, the lips retract showing the teeth, the tongue drops from the mouth and stays out.

The nostrils expand and the breathing becomes stertorous, the wings of the nostrils vibrate in a peculiar manner and tears roll from the eyes.

Along side of such a case we sometimes meet one that looks cheerful, eats and drinks well but shows weakness when put to work. He finely goes down exhibiting much the same symptoms as in the case first described; is raised with much difficulty and at first will make no effort to stand, but when fairly on his feet and well rubbed stands quite well and resumes feeding as before. While the animal is at rest, it takes an experienced person to detect anything wrong but while moving he will tumble down or tire out easily; a good type of the Jerseyman's "falling sickness," dying without showing any marked throat symptoms.

Again, in the next stall, we meet one that lies down and gets up, unaided to the last, but has almost complete paralysis of the tongue and muscles of deglutition, slowly developed, taking from a few hours to as many days to completely lose the power of swallowing. Such a case discharges much ropy saliva and the lining membrane of the oesophagus and trachea becomes putrid. The next horse shows weakness and in the course of a few days develop's facial palsy; the lower lip hanging pendulous and drawn to one side while the nose is usually drawn in the opposite direction giving him a ludicrous appearance. This horse has difficulty in picking up his food but swallows fairly well, he may have other and more grave symptoms added, or gradually recover, with the facial palsy, the only very noticeable trouble.

We next find a horse standing with a leg flexed and trembling, like a man with a palsied arm. He lies down, gets up, and moves about and eats well, apparently only inconvenienced by his palsied member. He is apt to become more generally affected and have to be helped up, but stands a good chance of recovery.

Now over here by the door in a drier part of the stable stands the sixth horse apparently all right, but when put to work has somewhat of a stiff gait, gets tired out and sweats easily. He recovers without difficulty if removed to new quarters.

These cases may all be seen together, but the first and second ones are most frequently met with. When we have it only in one horse of a severe type, it is general paralysis, with the throat the most affected. When we have it in an enzootic form confined to a certain valley, low lands or sea coast, it is of the second or spinal kind. Although going down flat on the side unable to turn up is a prominent symptom, at times we come across one that seems conscious that the recumbent position is the worst and most rapidly fatal one he can assume, and who keeps upon his legs, sweating, panting, trembling and rocking to and fro, until the last thread of strength is severed and sinks down to die in a few minutes. Much has been said of the spasms that seize them while in this recumbent position. That is an error. They remain conscious to the last or until advanced dissolution benumbs the brain. When first going down, they rest well for a short time, then become uncomfortable and after a few ineffectual attempts to turn upon the sternum, become much distressed and alarmed; and the spells of violent kicking and striking are only efforts to get away from the spot to which they are so thoroughly pinned. When such a spell is coming on he has only to be turned upon the sternum and all is over. The pathology of this disease is a highly interesting study.

From what has been observed as to character of pulse and temperature, we should not find any products of inflammation. In some of the most rapidly fatal cases, I have been unable to find any alteration of structure whatever. In others of some twenty-four hours duration, there is a frothy exudation into the air passages and congestion of the lungs, which arises from being in the prostrate position and from the muscles of respiration being partly paralyzed. These appearances have led many to conclude that they died of pneumonia, but there is no alteration of form or structure in the lungs. Quite early there is an exudate into the meninges of the spinal cord of a pale yellow color and in some instances of severe attack it is nearly red; but *the membranes themselves are not inflamed.* I consider this exudate a product of vaso motor paralysis and not of cerebro spinal meningitis, a highly inflammatory affection. This exudate may become so extensive as to cause pressure, increasing the primary paralysis and hastening death. In cases lasting from three to five days their appearances are well marked; and in those lasting from nine to fourteen days we find mortification of the mucous membrane of the throat and upper air passages and it may be detached or corroded away, threads of the tissue floating in an aqueous fluid, emitting a very offensive odor. This condition decreases as we follow the air passages down into the lungs, but even here are found patches of gangrenous tissue, the walls of air cells breaking and causing emphysema. This putrid condition pervades the whole system the flesh being soft, and easily torn with the fingers.

Now in all of the symptoms and pathological appearances we fail to find any evidence of an inflammatory affection; they all point to great prostration, paralysis and early dissolution, evidently the work of some depressent or poison to the nervous system. I wish the assignment to me of this subject had been deferred yet another summer and that I might have been able to give something more positive as to what the specific germ really is. I can only give theory and the reasons for the faith that it is a fungus, a mold, unlike other molds, in that it is poisonous to the Equine and Asinine species. Fungi grows upon dead and decaying organic bodies and on living plants. The mushroom and truffle are used as human food, while the toadstool, a first cousin, is a dangerous poison. Blight, mildew and rust are caused by a microscopic fungus and may not hurt a horse; while there is a kind that forms upon decaying vegetable matter *that kills* in the manner already described

Several years ago I noticed that brewers grains when kept until spoiled would give horses this complaint. A good illustration occurred about eight years ago when two car loads, in this condition, were sold at Hatfield and Doylestown Stations. There were forty-two deaths (that came to my notice) within a week's time, in barns into which this grain went *and not a single case anywhere else.* It is not necessary that the horse eats them but only that they be stored in the barn where the maturing and bursting spores fill the air, with myriads of germs which attach to and grow upon the hay and feed, which the horses eat. Turnips stored with the tops on, and cabbage leaves will sometimes produce the same result. In practice I frequently have a single case in a stable from yankee mangers; with slatted bottoms and want of proper vent below, a collection of hay and dirt under the manger had become moist and was undergoing decay.

The three horses which died suddenly, had a mash of boiled rye the evening before, which was completely covered with a mold.

In the early autumn of 1879, Fredrick Hanson, a tenant farmer lost his whole stock of horses and mules, five in number, inside of three days. I found that his oats had undergone fermentation from being stored before properly cured. I told him that they undoubtedly gave his horses the "choking distemper" and that he should use the remainder for cow feed. After procuring another team he concluded to take a load of these oats to the Philadelphia market. They went into three different stables and killed seventeen horses, all that ate of them. So I was informed by Hanson himself, who left the country from threatened prosecution.

In the fall of 1868 we had an enzootic of this disease, in a narrow valley, running east and west through Montgomery Township, starting at the head-waters and advancing steadily eastward about the fourth of a mile each day; driven by the prevailing west winds. It lasted two weeks and stopped short when the wind changed to the east. It was a mild form of the disease, eighty per cent. recovering. The larger half die on an average. It is a well established fact that horses of certain barns have this disease repeatedly while others in the same locality never have it. It usually occurs in damp barns and those in close proximity to meadows. Some of my clients have made alterations in and about their stables and afterwards escaped the dreaded malady. I have seen enough to thoroughly convince me that this disease originates from a species of fungus, and also that the microscope must furnish the aetiology. A healthy horse taken into an infected building for a short time, an hour or so, will contract the disease but a diseased one, taken into a healthy location will not carry infection, this fact suggests the most important point in treatment, namely, remove the patient to new quarters. Prevention is secured by avoiding the conditions that generate mold, and by discarding all food undergoing fermentation or putrefaction. The effects are best combatted by antiseptics and stimulents, internally and externally. I depend chiefly upon vinegar, chloride of sodium, camphor, capsicum, nux vomica, belladonna, mustard, good air, warm clothing and friction. It is also important to keep the patient upon his feet, except perhaps from a half to one hour daily, when the hoisting facilities are good.

